# Transcriptome analysis of *Cucumis sativus* infected by Cucurbit chlorotic yellows virus

**DOI:** 10.1186/s12985-017-0690-z

**Published:** 2017-02-02

**Authors:** Xinyan Sun, Zhenyue Wang, Qinsheng Gu, Honglian Li, Weili Han, Yan Shi

**Affiliations:** 1 College of Plant Protection, Henan Agricultural University, Zhengzhou, 450002 China; 2 Zhengzhou Fruit Research Institute, Chinese Academy of Agriculture Sciences, Zhengzhou, 450009 China

**Keywords:** CCYV, Transcriptome, Cucumber, RNA-Seq

## Abstract

**Background:**

*Cucurbit chlorotic yellows virus* (CCYV) is a recently reported bipartite crinivirus that causes chlorotic leaf spots and yellowing symptoms on the leaves of cucurbit plants. The virus–host interaction of CCYV remains to be elucidated, and the influence of criniviruses on the host gene transcriptome requires analysis.

**Methods:**

We used transcriptome sequencing to analyse the differentially expressed genes (DEGs) caused by CCYV infection.

**Results:**

CCYV infection resulted in 865 DEGs. The Kyoto Encyclopedia of Genes and Genomes (KEGG) pathway enrichment analysis identified 67 pathways, and the three major enrichment pathways (according to the *P*-values) were photosynthesis-antenna proteins (KO00196), phenylalanine metabolism (KO00360a), and phenylpropanoid biosynthesis (KO00940). Of the 13 DEGs identified in phenylalanine metabolism, 11 genes encode disease resistance-related phenylalanine ammonia-lyase (PAL) genes. Using quantitative real-time PCR, we validated the differential expression of 12 genes.

**Conclusions:**

Our study based on the CCYV–cucumber interaction provides comprehensive transcriptomic information, and will improve our understanding of host–crinivirus interactions.

**Electronic supplementary material:**

The online version of this article (doi:10.1186/s12985-017-0690-z) contains supplementary material, which is available to authorized users.

## Background

*Cucurbit chlorotic yellows virus* (CCYV) is a newly discovered cucurbit-infecting crinivirus of the family *Closteroviridae* [1–7]. CCYV causes chlorotic leaf spots and yellowing symptoms on the leaves of cucumber and melon, resulting in lower yields and poorer quality fruit. Like most members of the genus *Crinivirus*, CCYV consists of a bipartite positive sense RNA genome: 8607-nucleotide [nt] RNA1 and 8041-nt RNA2 [8]. RNA1 contains four open reading frames (ORFs): ORF1a, ORF1b, ORF2, and ORF3. ORF1a encodes methyltransferase and RNA helicase, while ORF1b encodes an RNA-dependent RNA polymerase motif. ORF2 and ORF3 encode P6 and P22, proteins with molecular masses of approximately 6 and 22 kDa, respectively. The 3′ ORFs of RNA1 are quite variable among the criniviruses; indeed, P6 and P22 show no significant similarity to corresponding proteins from other criniviruses [5]. RNA2 is predicted to encode eight proteins: P4.9, HSP70h, P6.5, P59, P9, CP, CPm, and P26. The genes encoding HSP70h, P59, CP, and CPm are conserved in the family *Closteroviridae* as a “hallmark gene array,” but the biological functions of the proteins encoded by RNA1 and RNA2 have yet to be elucidated. As a newly reported virus current studies were mainly focused on the establishment of detection method [9, 10], construction of infectious clone [11], and its transmission [12, 13].

Viral infection is a complex process involving an interaction between the virus and the host. Understanding host responses during viral infection will help in the development of effective strategies for virus control. Next-generation sequencing (NGS) technologies have enabled new approaches to transcriptome analysis [14, 15], and have been applied extensively to uncover the responses of plant hosts to viral infection [16–22].

This study used transcriptome analysis with a NGS approach to identify differentially expressed genes (DEGs) in cucumber after CCYV infection. The results showed that 865 genes were differentially expressed after CCYV infection, with 554 up-regulated and 311 down-regulated. Based on the *P*-values, the three major pathways involved were the photosynthesis-antenna protein (KO00196), phenylalanine metabolism (KO00360a) and phenylpropanoid biosynthesis (KO00940) pathways. The expression of genes involved in these three pathways was up-regulated. Using quantitative real-time PCR (qRT-PCR), we validated the differential expression of 12 genes. Our study will improve our understanding of plant–virus interactions.

## Methods

### Plant growth and virus inoculation

Cucumber plants (*Cucumis sativus*) xinyou36 were grown in a greenhouse under a 16 h light and 8 h dark cycle at 25 °C. A CCYV infectious clone was allowed to infiltrate cucumber cotyledons, as described previously [12]. Cucumber plants inoculated with a mock solution served as a control. At 18 days post-infiltration, the first leaf tissue was collected from CCYV- and mock-infected plants. Three biological replicates (three plants per replicate) were processed independently.

### RNA extraction

Total RNA was extracted from CCYV-infected leaves using TRIzol reagent according to the manufacturer’s instructions (Invitrogen, Carlsbad, CA, USA). The total RNA was treated with RNase-free DNase I (Takara Bio, Shiga, Japan) for 30 min at 37 °C to remove residual DNA. The RNA concentration was determined by micro-spectrophotometry analysis (NanoDrop 2000, Thermo Fisher Scientific, Waltham, MA, USA), and the RNA sample integrity was examined using Bio-analyzer 2100 equipment (Agilent Technologies, Germany).

### RNA-Seq library construction and sequencing

The extracted total RNA samples were used for cDNA synthesis. Poly (A) mRNA was isolated using oligo-dT beads (Qiagen, Hilden, Germany). The mRNA was broken into short fragments (~300 nt). First-strand cDNA was synthesized using random hexamer-primed reverse transcription. Second-strand cDNA was generated using RNase H and DNA polymerase I. The cDNA fragments were purified and washed for end repair and ligated to sequencing adapters. The cDNA fragments of suitable size were purified and enriched by PCR to obtain the final cDNA library. The cDNA library was sequenced using HiSeq™ 2500 equipment (Illumina, San Diego, CA, USA). The same equipment was also used for original image processing of sequences, base calling, and quality value calculations.

### Data analysis of RNA-Seq

Clean reads were selected after removing low-quality sequences (*i.e*., sequences in which more than 50% of the bases had a quality rating below 20), reads containing adaptor sequences, and reads with more than 5% unknown bases. Then, the sequencing reads were mapped to reference sequences using the Burrows–Wheeler Alignment Tool (http://bio-bwa.sourceforge.net/bwa.shtml). The read coverage of one gene was used to calculate the gene expression level, which was measured with the reads per kilobase of exon model per million mapped reads (RPKM) method.

### Evaluation of differentially expressed genes

After annotation, the expressed genes were compared between pairs of samples. The false discovery rate (FDR) was used to determine the *P*-value in multiple tests. For the analysis, we used FDR ≤ 0.001 and a log_2_ ratio ≥ 1 to assess the significance of gene expression differences.

To determine the main biological functions and pathways of the DEGs, all DEGs were mapped to terms in the Gene Ontology (GO) and Kyoto Encyclopedia of Genes and Genomes (KEGG) databases.

### Quantitative real-time PCR validation

To validate the transcriptome results, qRT-PCR was conducted using the total RNA for RNA-Seq. For reverse transcription, 1 μg of total RNA was used with the PrimeScript RT reagent kit (Takara), according to the manufacturer’s protocol. Twelve annotated unigenes were selected for validation. Primer sets were designed using Primer Premier software (ver. 5.0) (Table 1). The qRT-PCR reactions were performed with 5 μL of SYBR Green master mix, 20 ng of cDNA, and 200 nM each of the sense and antisense primers, in a total volume of 10 μL (Takara). Ubiquitin was used as a reference for calculating relative abundances using the 2^-△△CT^ method. All qRT-PCR experiments were performed in triplicate.

**Table 1:** Primers used for qRT-PCR

Gene name	Gene primer for qRT-PCR (5′ → 3′)
Csa1G009810	S^a^: ATTTGACCCGTTGGGATTAG
Csa1G009810	A^b^: GTTGACGACGAGGCGAAGTA
Csa3G099680	S^a^: GAGTTCCCTGGTGATTATGG
Csa3G099680	A^b^: TCTAAGACTTCGGGTGTTATG
Csa6G445760	S^a^: CCTCTTCGTGGAACCATTAC
Csa6G445760	A^b^: AACAATAGAAGCCAATCCTG
Csa3G645940	S^a^: CTACTACCTCCGACAACGC
Csa3G645940	A^b^: CACAGAGGCAGATTTCTCAT
Csa3G730800	S^a^: ATAATCACGCCAAGCCTCAG
Csa3G730800	A^b^: CACCGACACCGAACAATCCT
Csa3G836520	S^a^: GGCTTTCCAATAACAACACT
Csa3G836520	A^b^: ACCATTCGCCATATCTTCTG
Csa1G264550	S^a^: CCCCTTTGCTAAGGAACCTA
Csa1G264550	A^b^: TGTATGGCATCCCACTGTAT
Csa1G423100	S^a^: CCAACGGCCAAAGATTCTAC
Csa1G423100	A^b^: ATTGCAGCGATCATACTCGA
Csa2G179730	S^a^: AGGGAGGCGATAGTGGAATA
Csa2G179730	A^b^: GGCGGATAGTAATGACAGAACA
Csa2G295430	S^a^: ACGATGCGTGGGATGGGTAG
Csa2G295430	A^b^: AGCGTGGCAGGTTCCAGAAG
Csa6G217470	S^a^: CACCACCGCTGACACTGACT
Csa6G217470	A^b^: GGACGATGCTCGACCAAAG
Csa6G318690	S^a^: GAGCAGCCTCCAAGGATAAG
Csa6G318690	A^b^: TACGGACGCCAAGTTGTTAT
CsUBIF	S^a^: CACCAAGCCCAAGAAGATC
CsUBIR	A^b^: TAAACCTAATCACCACCAGC

^a^ *S* sense

^b^ *A* antisense

## Results

### Overview of transcriptome sequencing

To profile differential gene expression during CCYV infection, RNA-Seq libraries were constructed for the mock- and CCYV-inoculated cucumber plants. In total 29,823,892–39,138,040 clean reads of CCYV-infected cucumber plants, and 32,161,272–36,948,220 reads of mock-inoculated cucumber plants, were sequenced. After filtering, 26, 854,505–35,530,271 unique reads could be mapped to cucumber genes in the CCYV-infected cucumber plants, and 29,004,918–33,297,555 unique reads were mapped to cucumber genes in the mock-inoculated cucumber plants (Table 2).

**Table 2:** Summary of the transcriptome results

Library	CCYV-infected			Mock-inoculated		
	CCYV-1	CCYV-2	CCYV-3	CK-1	CK-2	CK-3
Total clean reads	34158314	39138040	29823892	32161272	36948220	33314552
High quality clean reads	33769294 (98.86%)	38661136 (98.78%)	29444160 (98.73%)	31754492 (98.74%)	36473130 (98.71%)	32946276 (98.89%)
Unique mapped reads	31165849 (92.37%)	35530271 (91.97%)	26854505 (91.28%)	29004918 (91.46%)	33297555 (91.40%)	30220259 (91.85%)
Number of mapped gene	17842 (76.75%)	18016 (77.49%)	17803 (76.58%)	17729 (76.26%)	17732 (76.27%)	17717 (76.21%)

Total clean reads: the raw data after sequencing

High-quality clean reads: the reads after filtering

Unique mapped reads: the high-quality clean reads that can be mapped to the cucumber genome

### Analysis of DEGs after CCYV infection

**Fig. 1:**
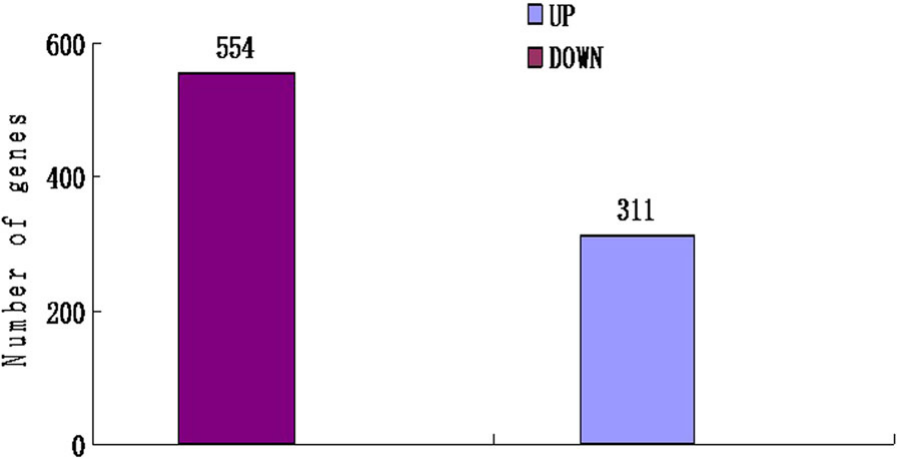
The number of differentially expressed genes (DEGs) in response to CCYV infection

An FDR ≤ 0.001 and log_2_ ratio ≥ 1 were used to identify DEGs. In total, 865 DEGs were identified, of which 311 were down-regulated and 554 were up-regulated in response to CCYV infection (Fig. 1). The fold change in gene expression was mainly between 1 and 2 (Additional file 1: Figure S1).

### GO analysis

**Fig. 2:**
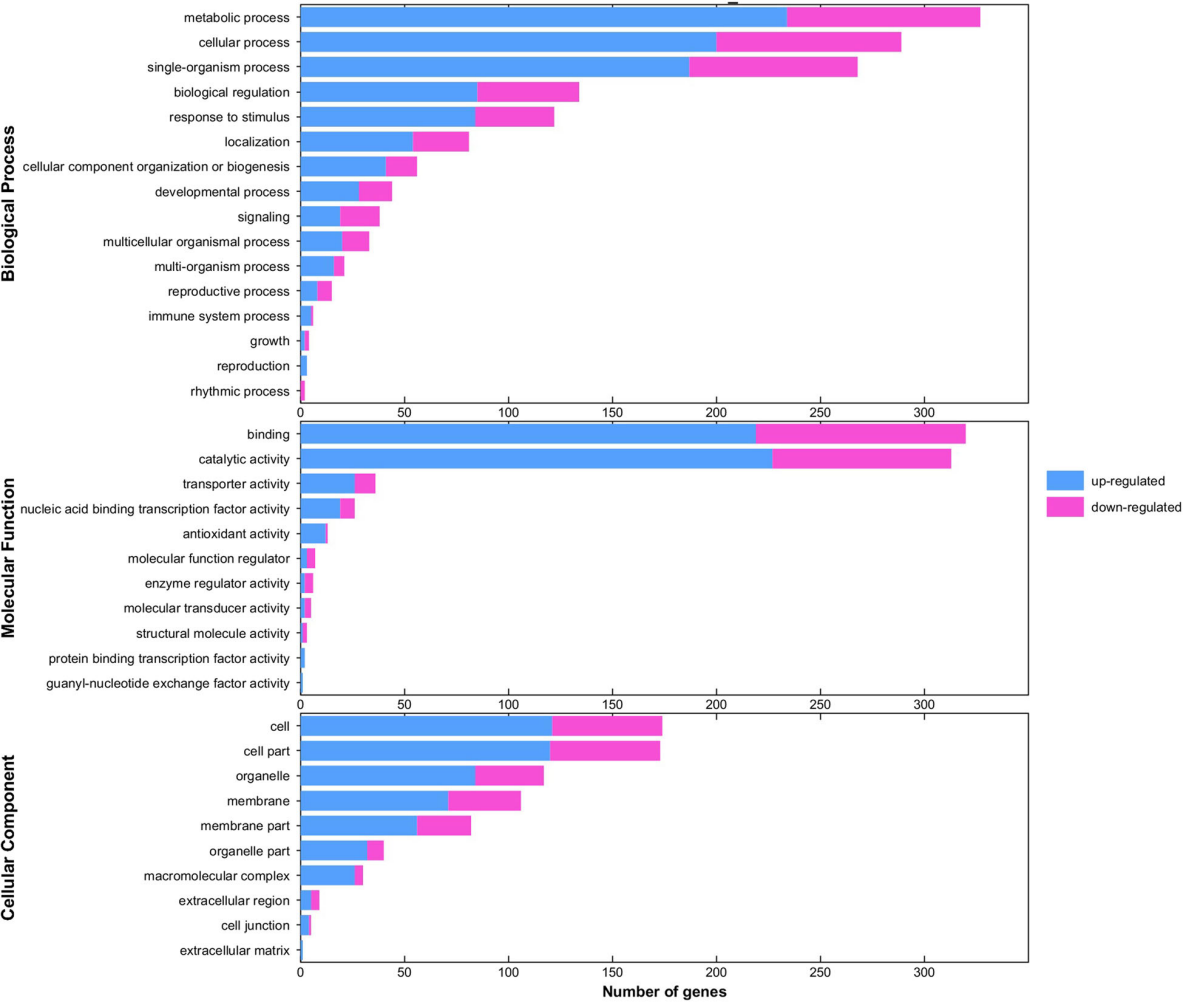
Gene ontology (GO) enrichment analysis of the DEGs. The genes were divided into three categories: cellular component, biological process, and molecular function genes

Further GO analyses of the DEGs classified the DEGs into 75 cellular component, 240 molecular function, and 755 biological process genes (Additional file 2: Table S1, Additional file 3: Table S2 and Additional file 4: Table S3). Of these, 11 cellular component, 18 molecular function, and 63 biological process genes were significant (Q < 0.05) (Additional file 5: Figure S2, Additional file 6: Table S4). The main categories identified for the cellular components, molecular functions, and biological processes were the cell and cell parts, binding and catalytic activity, and metabolic and cellular processes, respectively (Fig. 2).

### KEGG pathway enrichment analysis

**Fig. 3:**
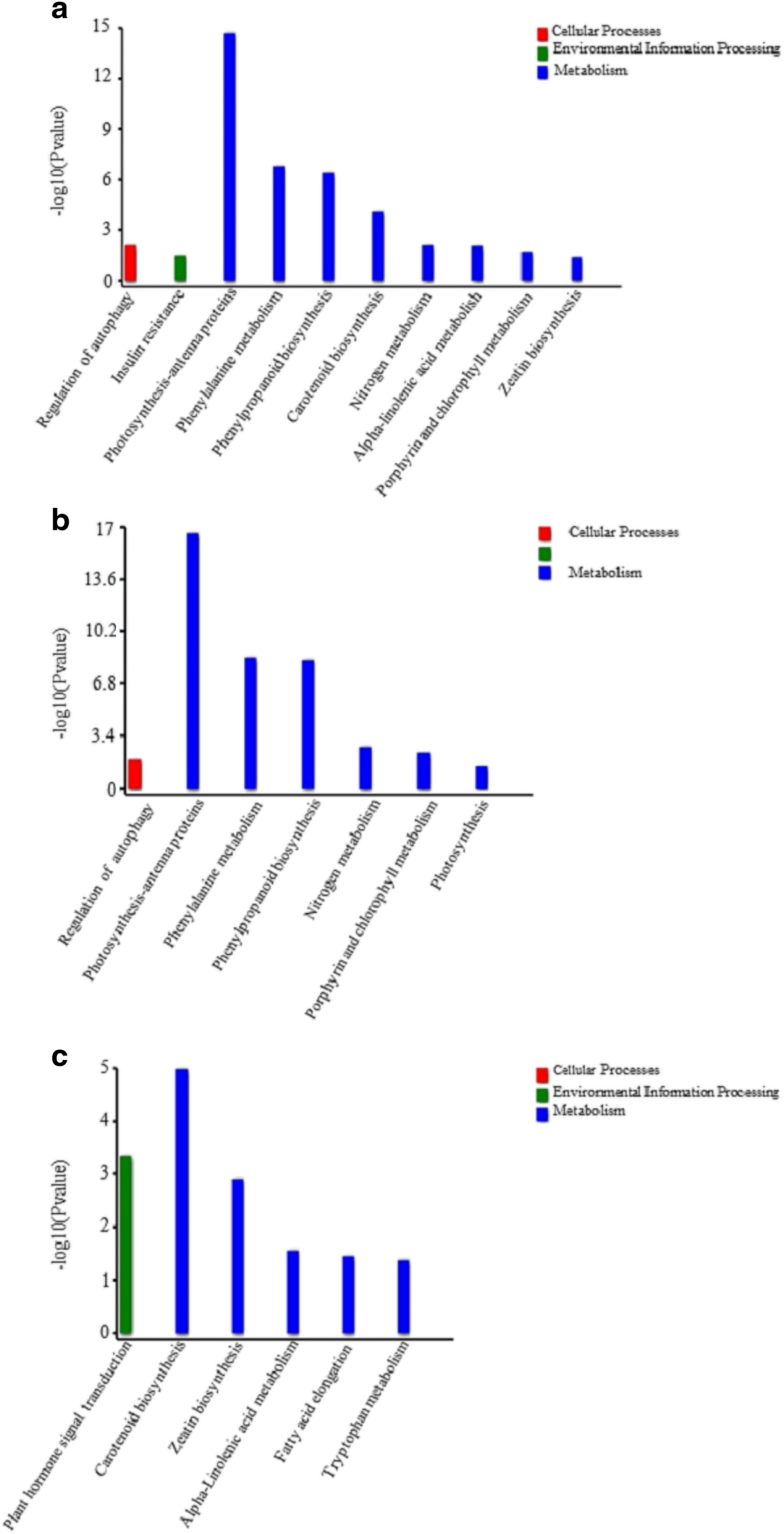
Kyoto Encyclopedia of Genes and Genomes (KEGG) pathway enrichment analyses. **a** All pathways were enriched using the criterion *P* < 0.05; **b** Pathway enrichment analysis of up-regulated genes. **c** Pathway enrichment analysis of down-regulated genes

To further understand the molecular and biological functions of the DEGs, the genes were mapped to the KEGG database. Pathway enrichment analysis identified 67 pathways, of which 10 were enriched using the criterion *P* < 0.05 (Fig. 3a, Additional file 7: Figure S3). Further enrichment analysis of up- and down-regulated genes showed that genes involved in photosynthesis-antenna protein synthesis, phenylalanine metabolism, phenylpropanoid biosynthesis, nitrogen metabolism, porphyrin and chlorophyll metabolism, photosynthesis, and the regulation of autophagy were up-regulated (Fig. 3b), while genes involved in carotenoid biosynthesis, plant hormone signal transduction, zeatin biosynthesis, alpha-linolenic acid metabolism, fatty acid elongation, and tryptophan metabolism were down-regulated (Fig. 3c). Of the 13 identified phenylalanine metabolism DEGs, 11 genes encode phenylalanine ammonia-lyase (PAL) genes, which are associated with disease resistance.

### Validation of the transcriptome data by qRT-PCR

**Fig. 4:**
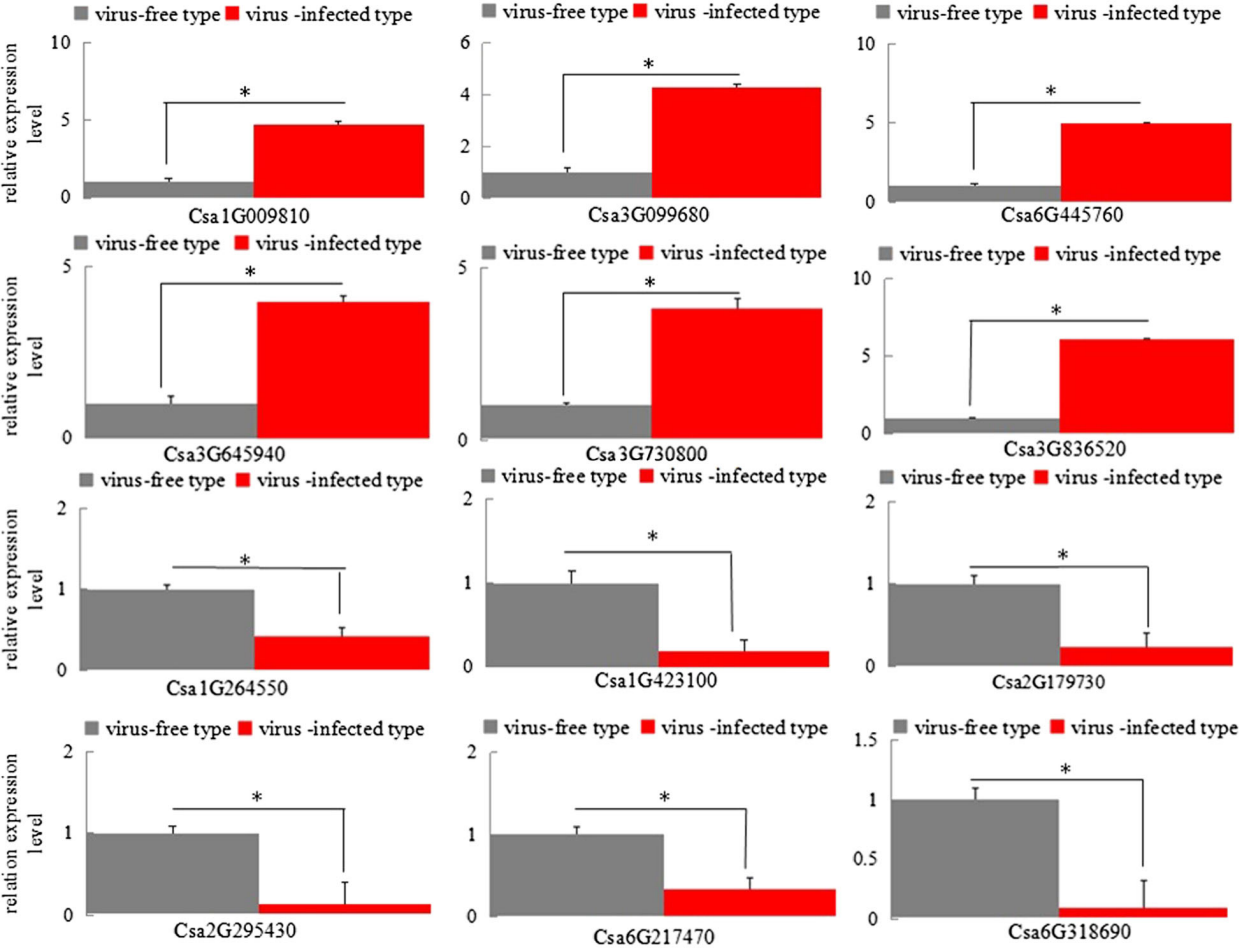
Quantitative real-time PCR (RT-PCR) validation of DEGs. The cucumber ubiquitin gene was used as an internal control. *Error bars* represent the standard deviation of the qRT-PCR signals (*n* = 3). *Asterisks* indicate statistically significant differences compared with the control (*P* < 0.05)

To validate the DEGs, 12 unigenes were selected for qRT-PCR analysis: four unigenes from the top 10 pathways with the most significant differences, and eight unigenes selected randomly from other pathways. The results indicated that the qRT-PCR data were consistent with the transcriptome results (Fig. 4).

## Discussion

The recently reported CCYV virus can reduce cucurbit quality and yield, and is become increasingly important worldwide [23]. In this study, we used NGS approaches to investigate the DEGs associated with CCYV infection in cucumber plants. RNA-Seq analysis identified 19,192 known, and 532 new genes compared with the existing 23,248 cucumber reference genes, of which 865 genes were differentially expressed. KEGG pathway enrichment analysis revealed that photosynthesis-antenna protein pathway, phenylalanine metabolism, and phenylpropanoid biosynthesis showed the most significant difference based on the Q-value. Further analysis of the top 10 most significantly enriched KEGG pathways showed that 8 out of 10 were related to metabolism, while the other two regulated autophagy and insulin resistance.

Phenylpropanoids play important roles in plant responses towards biotic and abiotic stress [24, 25]. Phenylalanine ammonia-lyase (PAL) contributes to several pathways including phenylpropanoid biosynthesis and is an important interface between primary and secondary metabolism [24, 26]. PAL plays an important role in plant defence, and *PAL* gene expression was upregulated in response to different pathogen infection [27–29]. Here we found that 11 DEGs encoding *PAL* gene were upregulated implying the involvment of *PAL* gene in the defense against CCYV infection. In our study genes involved in the “plant-pathogen interaction” pathway were up-regulated during CCYV infection. These defense-related genes included WRKY transcription factors, calcium binding protein, and respiratory burst oxidase homolog protein. Here seven cucumber WRKY transcription factors were found to be induced after CCYV infection. Previous studies have shown that various WRKY genes from different plants are induced in response to the infection by bacterial [30], fungal [31–33], and viral pathogens [34, 35].

The repression of genes related to photosynthesis has been reported in chlorotic tissues [36–40]. Here, we found that genes involved in pathways related to photosynthesis were up-regulated. This difference may be due to the time point at which the samples were collected, because *Pepino mosaic virus* strongly repressed photosynthesis-related genes 4 days post inoculation, while several genes involved in chlorophyll binding and light harvesting were induced at 12 days post inoculation [41]. Besides, In Arabidopsis leaves infected with fungi *Albugo candida* and in tomato plants infected with *Botrytis cinerea*, enhanced photosynthesis was oberved surrounding the area with decreased photosynthesis at the infection site [42]. The stimulation of photosynthesis maybe due to the defence strategy of the plant.

## Conclusion

Using transcriptome sequencing, we obtained a genome-wide transcript profile of cucumber plants infected by CCYV, and genes involved in the plant defense system were found to be differentially expressed after CCYV infection. The information obtained in this study will help investigations of the detailed mechanisms of the CCYV–host interaction and could identify resistance genes.

## Additional files

Additional file 1: Figure S1. Volcano plot of the transcriptome results. Red points represent up-regulation, green points represent down-regulation, and black points represent no difference. (JPG 59 kb)
Additional file 2: Table S1. List of DEGs classified as cellular component genes. (XLS 33 kb)
Additional file 3: Table S2. List of DEGs classified as molecular function genes. (XLS 4 kb)
Additional file 4: Table S3. List of DEGs classified as biological process genes. (XLS 4 kb)
Additional file 5: Figure S2. Significantly enriched GO terms. (JPG 6565 kb)
Additional file 6: Figure S3. Pipeline for GO annotation. (JPG 24 kb)
Additional file 7: Table S4. Ten Significantly enriched pathways. (DOC 37 kb)
