# Supplementary figures and images for: Transcriptome analysis of Cucumis sativus infected by Cucurbit chlorotic yellows virus
Source: Virol J. 2017 Feb 2;14:18. doi: 10.1186/s12985-017-0690-z (PMC5288851; doi:10.1186/s12985-017-0690-z)

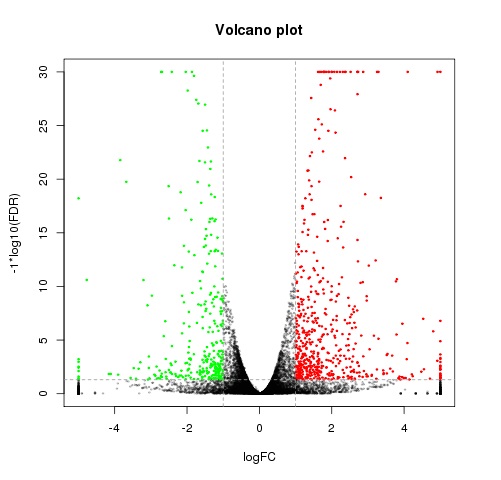

Supplement: Additional file 1: Figure S1. — Volcano plot of the transcriptome results. Red points represent up-regulation, green points represent down-regulation, and black points represent no difference. (JPG 59 kb) [file 12985_2017_690_MOESM1_ESM.jpg]

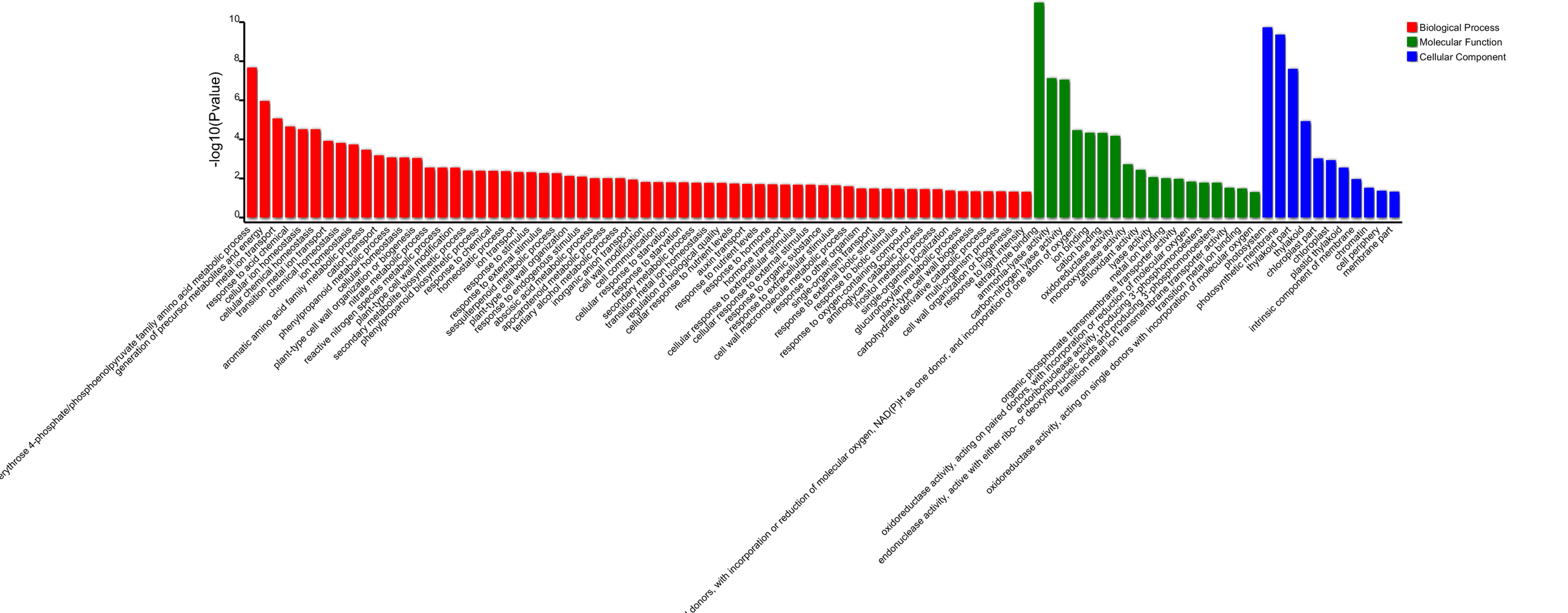

Supplement: Additional file 5: Figure S2. — Significantly enriched GO terms. (JPG 6565 kb) [file 12985_2017_690_MOESM5_ESM.jpg]

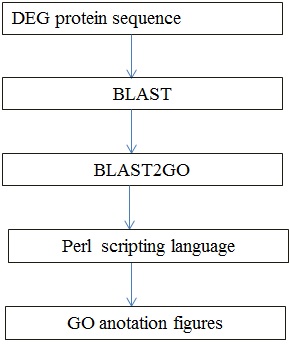

Supplement: Additional file 6: Figure S3. — Pipeline for GO annotation. (JPG 24 kb) [file 12985_2017_690_MOESM6_ESM.jpg]
