# Supplementary material for: Transcriptome analysis of Cucumis sativus infected by Cucurbit chlorotic yellows virus
Source: Virol J. 2017 Feb 2;14:18. doi: 10.1186/s12985-017-0690-z (PMC5288851; doi:10.1186/s12985-017-0690-z)
Supplement: Additional file 7: Table S4. — Ten Significantly enriched pathways. (DOC 37 kb) [file 12985_2017_690_MOESM7_ESM.doc]

| **Pathway** | **DEGs genes with pathway annotation (162)** | **Pathway ID** |
| --- | --- | --- |
| [Photosynthesis - antenna proteins](../../../../E:%5C%E5%AD%A6%E4%B9%A0%5C%E5%85%AC%E5%8F%B8%E5%90%88%E5%90%8C%5C%E6%9C%80%E6%96%B0GDR1283-Cucumis_sativus-6-RNAseq-result%5C7.GroupsDifferentialExpression%5CEnrichment%5CKO%5CCsCK-vs-CsCCYV_A.htm#gene1%23gene1) | 13 (8.02%) | ko00196 |
| [Phenylalanine metabolism](../../../../E:%5C%E5%AD%A6%E4%B9%A0%5C%E5%85%AC%E5%8F%B8%E5%90%88%E5%90%8C%5C%E6%9C%80%E6%96%B0GDR1283-Cucumis_sativus-6-RNAseq-result%5C7.GroupsDifferentialExpression%5CEnrichment%5CKO%5CCsCK-vs-CsCCYV_A.htm#gene2%23gene2) | 13 (8.02%) | ko00360 |
| [Phenylpropanoid biosynthesis](../../../../E:%5C%E5%AD%A6%E4%B9%A0%5C%E5%85%AC%E5%8F%B8%E5%90%88%E5%90%8C%5C%E6%9C%80%E6%96%B0GDR1283-Cucumis_sativus-6-RNAseq-result%5C7.GroupsDifferentialExpression%5CEnrichment%5CKO%5CCsCK-vs-CsCCYV_A.htm#gene3%23gene3) | 22 (13.58%) | ko00940 |
| [Carotenoid biosynthesis](../../../../E:%5C%E5%AD%A6%E4%B9%A0%5C%E5%85%AC%E5%8F%B8%E5%90%88%E5%90%8C%5C%E6%9C%80%E6%96%B0GDR1283-Cucumis_sativus-6-RNAseq-result%5C7.GroupsDifferentialExpression%5CEnrichment%5CKO%5CCsCK-vs-CsCCYV_A.htm#gene4%23gene4) | 7 (4.32%) | ko00906 |
| [Regulation of autophagy](../../../../E:%5C%E5%AD%A6%E4%B9%A0%5C%E5%85%AC%E5%8F%B8%E5%90%88%E5%90%8C%5C%E6%9C%80%E6%96%B0GDR1283-Cucumis_sativus-6-RNAseq-result%5C7.GroupsDifferentialExpression%5CEnrichment%5CKO%5CCsCK-vs-CsCCYV_A.htm#gene5%23gene5) | 5 (3.09%) | ko04140 |
| [Nitrogen metabolism](../../../../E:%5C%E5%AD%A6%E4%B9%A0%5C%E5%85%AC%E5%8F%B8%E5%90%88%E5%90%8C%5C%E6%9C%80%E6%96%B0GDR1283-Cucumis_sativus-6-RNAseq-result%5C7.GroupsDifferentialExpression%5CEnrichment%5CKO%5CCsCK-vs-CsCCYV_A.htm#gene6%23gene6) | 5 (3.09%) | ko00910 |
| [alpha-Linolenic acid metabolism](../../../../E:%5C%E5%AD%A6%E4%B9%A0%5C%E5%85%AC%E5%8F%B8%E5%90%88%E5%90%8C%5C%E6%9C%80%E6%96%B0GDR1283-Cucumis_sativus-6-RNAseq-result%5C7.GroupsDifferentialExpression%5CEnrichment%5CKO%5CCsCK-vs-CsCCYV_A.htm#gene7%23gene7) | 7 (4.32%) | ko00592 |
| [Porphyrin and chlorophyll metabolism](../../../../E:%5C%E5%AD%A6%E4%B9%A0%5C%E5%85%AC%E5%8F%B8%E5%90%88%E5%90%8C%5C%E6%9C%80%E6%96%B0GDR1283-Cucumis_sativus-6-RNAseq-result%5C7.GroupsDifferentialExpression%5CEnrichment%5CKO%5CCsCK-vs-CsCCYV_A.htm#gene8%23gene8) | 5 (3.09%) | ko00860 |
| [Insulin resistance](../../../../E:%5C%E5%AD%A6%E4%B9%A0%5C%E5%85%AC%E5%8F%B8%E5%90%88%E5%90%8C%5C%E6%9C%80%E6%96%B0GDR1283-Cucumis_sativus-6-RNAseq-result%5C7.GroupsDifferentialExpression%5CEnrichment%5CKO%5CCsCK-vs-CsCCYV_A.htm#gene9%23gene9) | 5 (3.09%) | ko04931 |
| [Zeatin biosynthesis](../../../../E:%5C%E5%AD%A6%E4%B9%A0%5C%E5%85%AC%E5%8F%B8%E5%90%88%E5%90%8C%5C%E6%9C%80%E6%96%B0GDR1283-Cucumis_sativus-6-RNAseq-result%5C7.GroupsDifferentialExpression%5CEnrichment%5CKO%5CCsCK-vs-CsCCYV_A.htm#gene10%23gene10) | 3 (1.85%) | ko00908 |
